# Epilepsy in rural South Africa: Patient experiences and healthcare challenges

**DOI:** 10.1002/epi4.12999

**Published:** 2024-06-17

**Authors:** Lufuno Makhado, Angelina Maphula, Richard Teke Ngomba, Ofhani Prudance Musekwa, Thendo Gertie Makhado, Muofheni Nemathaga, Mukovhe Rammela, Muimeleli Munyadziwa, Pasquale Striano

**Affiliations:** ^1^ Department of Public Health, Faculty of Health Sciences University of Venda Thohoyandou Limpopo Province South Africa; ^2^ Department of Psychology, Faculty of Health Sciences University of Venda Thohoyandou Limpopo Province South Africa; ^3^ School of Pharmacy University of Lincoln, Joseph Banks Laboratories Lincolnshire UK; ^4^ Department of Psychology, Faculty of Humanities University of Johannesburg Johannesburg South Africa; ^5^ Department of Advanced Nursing Sciences, Faculty of Health Sciences University of Venda Thohoyandou Limpopo Province South Africa; ^6^ Pediatric Neurology and Muscular Diseases Unit IRCCS Istituto Giannina Gaslini Genoa Italy; ^7^ Department of Neurosciences, Rehabilitation, Ophthalmology, Genetics, Maternal and Child Health University of Genova Genoa Italy

**Keywords:** antiseizure medication, epilepsy, people with epilepsy, seizures, side effects

## Abstract

**Objective:**

This study investigates the prevalent issues of healthcare access and the impact of antiseizure treatments among people with epilepsy (PWE) in rural Limpopo and Mpumalanga, South Africa, where healthcare facilities and affordable treatments are often inadequate.

**Methods:**

Using a cross‐sectional survey, 162 PWE were selected using multistage sampling across the provinces. Data were collected via a structured questionnaire and analyzed descriptively using SPSS v27.

**Results:**

Most of the participants experienced seizures intermittently, with 70.6% in Limpopo and 53.3% in Mpumalanga reporting occasional episodes, whereas a significant minority in both regions—20.6% and 40%, respectively—suffered from frequent seizures. A notable portion of PWE also reported recurring side effects from antiseizure drugs, which led to consequential life disruptions, including educational dropout and unemployment.

**Significance:**

The findings underscore an urgent need for enhanced educational programs and increased awareness to improve the understanding and management of epilepsy in these underserved areas. Optimizing care for PWE requires a multifaceted approach, including evaluating healthcare accessibility, affordability, and societal beliefs influencing treatment adherence. The study advocates for government and policy interventions to mitigate the quality of life deterioration caused by epilepsy and its treatment in rural communities.

**Plain Language Summary:**

In Limpopo and Mpumalanga, many individuals with epilepsy experience seizures occasionally, while a significant minority have them frequently. Numerous people also suffer recurring side effects from antiseizure medications, impacting their lives severely by causing school dropouts and job losses. This underscores the urgent need for improved education and awareness programs to manage epilepsy in these provinces effectively. The study urges government action and policy reforms to enhance care and support for people with epilepsy in rural areas, aiming to improve their quality of life.

**Key points**

- *Epilepsy insights*: The study explored epilepsy experiences with treatment. Diagnosis often occurs in childhood. Seizures and effects vary widely.
- *Treatment complexities*: Seizures persist despite meds, leading to diverse symptoms. Side effects impact daily life and interactions.
- *Social challenges*: Many PWE are unmarried due to epilepsy burdens. Isolation, dependency, and discrimination are prevalent.
- *Actionable takeaways*: Diagnose accurately, improve seizure control, and address treatment effects. Support systems are crucial. Awareness and research are needed.

## 1 INTRODUCTION

Approximately 50 million people globally live with epilepsy, accounting for ~ 0.5% of the total global disease burden and 13 million disability‐adjusted life years, affecting about one in 10 individuals.^1^ Currently, there is no cure for epilepsy, with medication only controlling symptoms, leaving many patients with treatment‐resistant epilepsy.^2^ An estimated 15 million people aged 30–69 die from non‐communicable diseases (NCDs) like epilepsy.^3^ Epilepsy increases the risk of early death,^4^ including sudden unexpected death in epilepsy (SUDEP) and status epilepticus.^3^ Neurological disorders cause about 9% of worldwide deaths, excluding multiple disabilities and injuries.^5^, ^6^

In low‐ and middle‐income countries (LMICs), PWE faces higher risks of poor health outcomes, reduced quality of life, and premature death, often due to the country's developmental and economic status. Despite being a common NCD, epilepsy is often overlooked by health practitioners and researchers in LMICs. Kwon et al.^2^ highlighted a significant global epilepsy treatment gap (ETG), with Owolabi et al.^7^ revealing a 69% ETG in Sub‐Saharan Africa, predominantly in rural areas. Wagner^8^ reported a 63% ETG in rural South Africa, attributed to widespread rural settlements and economic instability.

Access to healthcare and affordable treatment is a significant challenge in LMICs, with over 75% of epilepsy patients lacking access to effective and low‐cost antiseizure medications (ASMs).^7^ First‐line antiseizure drugs cause adverse side effects in nearly half of the patients undergoing treatment.^9^ Drug resistance is observed in 32% of epilepsy patients.^10^, ^11^ Early initiation of ASMs after the first seizure has been shown to reduce the risk of a second seizure.^12^ Table 1 highlights key features of common antiseizure drugs in South Africa.

**TABLE 1:** Characteristics of widely used first‐generation antiseizure drugs for treating epilepsy in South Africa.

Drug	Mechanism of action	Approved used (FDA, EMA)	Main uses	Main limitations
Phenobarbital	GABA potentiation	Partial and generalized convulsive seizures, sedation, anxiety, and sleep disorder	Focal and generalized seizures, the most cost‐effective treatment for epilepsy	Enzyme inducer, which is not useful in the absence of seizures, is less effective than carbamazepine or phenytoin for focal seizures in mostly new‐onset epilepsy
Carbamazepine	Na^+^ Channel blocker	Partial and generalized convulsive seizures, trigeminal pain, bipolar disorder	First‐line drug for focal and generalized seizures with focal onset.	Enzyme inducer, not useful for absence or myoclonic seizures
Valproate (Epilim)	Sodium channel and T‐type calcium channel blocker.	Partial and generalized convulsive seizures and migraine prophylaxis	The first line for focal and generalized seizures, none of the new drugs is more effective than valproate	An enzyme inhibitor, substantial teratogenicity, weight gain
Clonazepam	GABA potentiation	Myoclonic seizures and panic disorders	Focal and generalized seizure, no clinical hepatotoxicity	Substantial sedative tolerance (loss of efficacy) is available only for adjunctive use
Diazepam	GABA potentiation	Convulsive disorder, epilepsy, anxiety, alcohol, withdrawal.	Intravenous use for focal and generalized seizures, no skin hypersensitivity	Mainly used during an emergency only and currently for adjunctive

Despite treatment challenges, South Africa has access to affordable options like Phenobarbital, costing $5–$10 annually. Phenobarbital tablets are widely accessible in most countries.^13^ However, its use is limited in developed nations due to a lower tolerance than carbamazepine or phenytoin.^14^ Phenobarbital is preferred in large‐scale community programs, especially in remote areas.^15^, ^16^ The availability of ASMs differs across income levels in first‐, second‐, and third‐generation ASMs.^13^ Tsvere et al.^17^ highlighted the increased risk of bone fractures in women with epilepsy due to seizures and certain ASMs, emphasizing the need for effective, safe, and convenient ASMs.

In LMICs, minimal research on epilepsy and poor resource management contribute to a lack of understanding and defining the condition. Misdiagnosis, lack of treatment, and low medication adherence are ongoing issues in South Africa.^4^, ^17^, ^18^, ^19^, ^20^, ^21^ Despite this, no studies have focused on epilepsy experiences in Limpopo or Mpumalanga, including treatment effects on PWE. This gap in research contributes to the low quality of life for PWE and slows efforts toward improving epilepsy management and treatment. Kwon et al.^2^ found a worldwide ETG, with about 70% in LMICs. The lack of neurologists may also contribute to the high treatment gap.^17^, ^22^, ^23^, ^24^ In areas with fewer clinical specialists, there is a higher likelihood of neglecting those in need of epilepsy treatment. The current study aims to describe epileptic seizures, including frequency and severity, the efficacy of treatment and its adverse effects in rural South Africa.

## 2 MATERIALS AND METHODS

### 2.1 Study design

This study adopted a quantitative approach using a cross‐sectional survey study. This approach was selected because of its ability to collect large amounts of data, strengthening the study's objectivity. The study was conducted from February to December 2021.

### 2.2 Study setting

This study was conducted in selected rural communities of the Limpopo and Mpumalanga Provinces. The communities chosen in Limpopo included Malavuwe/Nweli, Mtititi, and Bochum, whereas the communities selected in Mpumalanga included Clara, Jerusalem, and Akornhoek. These communities were selected based on cultural diversity and the high prevalence of epilepsy.

### 2.3 Study subjects

The study included community members who had lived in the selected villages of the Limpopo and Mpumalanga provinces for at least six months or more. Furthermore, those aged 18–59 years, literate and illiterate, were included in the study.

### 2.4 Sample size and procedure

The sample size for the study was determined using an online Raosoft sample size calculator with a confidence level set at 95% and a margin of error of 5%. This yielded a target sample size of 169 participants out of 300 PWE. Of 169, only 162 fully completed questionnaires were included in this study's analysis. We used a non‐probability purposive sampling approach to select specific provinces and villages where PWE resides. Within the selected provinces, a multistage sampling technique was applied. The first stage involved choosing sub‐districts with the highest rates of PWE. The subsequent stages entailed selecting specific villages and PWE. All PWE residing in the selected village were eligible for study participation.

### 2.5 Data collection instrument

A research assistant–administrator questionnaire was used to collect data. The questionnaire included demographic characteristics, clinical variables, and outcome variables on epilepsy treatment and practice, with 15 questions on treating epilepsy and practices toward it. These proxy questions were closed‐ended, and the responses were ranked as follows: Never = 0, Sometimes = 1, Always = 2.

### 2.6 Data quality assurance

The questionnaire used in the study was initially written in English and later translated into local languages such as Tshivenda, Tsonga, Northern Pedi, and SiSwati. Language professionals were involved in the translation process, and back translation was conducted to ensure accuracy. Data were collected by home‐based carers and community health care workers in Limpopo and Mpumalanga provinces. Before the data collection, a pretest was conducted with 10% of the participants, and the questionnaire was modified based on the results. The research team monitored the data collectors daily, and supervisors and investigators reviewed the completed questionnaires for accuracy. Some content expressions were also rewritten to make them more understandable for the research participants.

### 2.7 Data analysis procedure

The data collected was analyzed using both descriptive and inferential statistics. Descriptive statistics involves calculating frequencies, percentages, means, and standard deviations to summarize the data. These measures provided an overview of the demographic characteristics of the sample, as well as the distribution of responses to survey items. We used Chi‐square, Phi and Crammer's *V* tests for inferential analysis to establish differences between the provinces and evaluate the associations between PWE responses. These tests were selected based on the data type, mainly nominal and ordinal. The level of significance was set at *p* < 0.05 for all tests. All analyses used Statistical Packaging for Social Sciences (SPSS) version 27.

### 2.8 Ethical considerations

Ethical considerations were followed throughout the study. Permission was sought from the Department of Advanced Nursing Science for quality assurance, the Faculty of Health for quality assurance and approval, The University of Venda Research Ethics Committee, the Limpopo and Mpumalanga Department of Health Research Committee, the Royal Councils and the participants. Informed consent was obtained from the participants before data collection. The researchers adhered to confidentiality, respect, privacy, and anonymity.

## 3 RESULTS

### 3.1 Socio‐demographic characteristics of participating PWE

About 162 PWE from Limpopo (n = 102) and Mpumalanga (n = 60) provinces participated in the study. The ages of the PWE included were PLWE ≥18 years old, with most participants aged between 18 and 45 years. Most PWE were males in Limpopo (39.2%) and Mpumalanga (46.7%) provinces. The study's Pedi, Swati, Venda and Tsonga ethnicities were well represented, as indicated in Table 2.

**TABLE 2:** Socio‐demographic characteristics for PWE.

	Province		*χ* ^2^ (*p*‐value)
	LP (*n* = 102)	MP (*n* = 60)	
Age			
18–25 years	25 (24.5%)	19 (31.7%)	2.425 (0.658)
26–35 years	21 (20.6%)	15 (25.0%)	
36–45 years	23 (22.5%)	9 (15.0%)	
46–55 years	14 (13.7%)	8 (13.3%)	
56 years and above	19 (18.6%)	9 (15.0%)	
Gender			
Female	40 (39.2%)	28 (46.7%)	2.747 (0.253)
Male	62 (60.8%)	31 (51.7%)	
Other	0 (0.0%)	1 (1.7%)	
Ethnicity			
Pedi	83 (81.4%)	1 (1.7%)	132.033 (< 0.001)
Swati	0 (0.0%)	15 (25.0%)	
Venda	11 (10.8%)	0 (0.0%)	
Tsonga	7 (6.9%)	42 (70.0%)	
Afrikaans	0 (0.0%)	1 (1.7%)	
Zulu	0 (0.0%)	1 (1.7%)	
Sotho	1 (1.0%)	0 (0.0%)	
Education			
None	34 (33.3%)	17 (28.3%)	6.046 (0.196)
Primary	38 (37.3%)	32 (53.3%)	
Secondary (no matric)	21 (20.6%)	10 (16.7%)	
Secondary (matric)	8 (7.8%)	1 (1.7%)	
Tertiary	1 (1.0%)	0 (0.0%)	
Religion			
Christianity	59 (57.8%)	50 (83.3%)	11.468 (0.003)
Traditional	41 (40.2%)	10 (16.7%)	
Islam	2 (2.0%)	0 (0.0%)	
Employment status			
Not employed	95 (93.1%)	56 (93.3%)	1.627 (0.443)
Self‐employed	1 (1.0%)	2 (3.3%)	
Employed	6 (5.9%)	2 (3.3%)	
Marital status			
Not married	74 (72.5%)	44 (73.3%)	22.720 (< 0.001)
Married	22 (21.6%)	2 (3.3%)	
Divorced	1 (1.0%)	2 (3.3%)	
Widow/widower	3 (2.9%)	3 (50%)	
Cohabitation	2 (2.0%)	9 (15.0%)	
Age when first diagnosed with epilepsy			
0–9 years	37 (36.3%)	35 (58.3%)	10.436 (0.064)
10–19 years	28 (27.5%)	15 (25.0%)	
20–29 years	14 (13.7%)	6 (10.0%)	
30–39 years	13 (12.7%)	2 (3.3%)	
40–49 years	4 (3.9%)	1 (1.7%)	
50 years and above	6 (5.9%)	1 (1.7%)	
Distance from residence to the nearest healthcare facility or clinic			
0–9 km	33 (32.4%)	49 (81.7%)	39.621 (< 0.001)
10–19 km	50 (49.0%)	4 (6.7%)	
20–29 km	11 (10.8%)	4 (6.7%)	
30–39 km	8 (7.8%)	3 (5.0%)	
Distance from residence to the nearest hospital			
0–9 km	4 (3.9%)	6 (10.0%)	42.252 (< 0.001)
10–19 km	43 (42.2%)	4 (6.7%)	
20–29 km	25 (24.5%)	24 (40.0%)	
30–39 km	21 (20.6%)	9 (15.0%)	
40–49 km	2 (2.0%)	15 (25.0%)	
50 km and above	7 (6.9%)	2 (3.3%)	

Most PWE had no formal or only primary education, meaning they did not go to school or only attended the foundational phase of primary school. In Limpopo, 33.3% of participants had no education, 37.3% had primary education, 20.6% had secondary education without Matric, 7.8% had completed Matric, and 1.0% had tertiary education. In contrast, in Mpumalanga, 28.3% of participants had no education, 53.3% had primary education, 16.7% had secondary education without Matric, 1.7% had completed Matric, and none had tertiary education. Thus, the illiteracy rate is 70.6% in Limpopo province and 81.3% in Mpumalanga province. Christianity (57.8% and 83.3%) was most PWE's religion, followed by traditional beliefs (40.2% and 16.7%) in Limpopo and Mpumalanga provinces. Moreover, most PWE are not married (LP = 72.5%; MP = 73.3%) and are not employed (LP = 93.1%; MP = 93.3%). There was a statistical difference between PWE in Limpopo and Mpumalanga province when it comes to distance to PHC (*x* ^2^ = 39.621^a^; *p* > 0.000) and Hospital facilities (*x* ^2^ = 42.252; *p* > 0.000), whereas there was no statistical difference concerning the age when diagnosed (*x* ^2^ = 10.436; *p* = 0.064).

### 3.2 Experiences of epileptic seizures and treatment‐related effects

Most PWE reported that they sometimes experience seizures (70.6% and 53.3%); however, some PWE always experienced seizures (20.6% and 40%) in Limpopo and Mpumalanga provinces, respectively. This study revealed significant differences in the experiences of an aura or warning before an epileptic seizure (*x* ^2^ = 15.570; *p* ≥ 0.000; Phi and Crammers = 0.308), as indicated in Table 3. It was also provided through these results that most PWE could sometimes feel (LP = 35%; MP = 30%) and always (LP = 45.1%; MP = 50%) that they were going to have an epileptic seizure and there were no statistical differences between the two provinces.

**TABLE 3:** Experiences of seizures among PWE.

Crosstab on frequency of epileptic seizures			
	Province		*χ* ^2^ (*p*‐value) Phi and Cramer's
	Limpopo	Mpumalanga	
Frequency of seizures			
Never	9 (8.8%)	4 (6.7%)	7.096 (0.029) (0.209)
Sometimes	72 (70.6%)	32 (53.3%)	
Always	21 (20.6%)	24 (40.0%)	
Have an aura/warning before an epileptic seizure			
Never	7 (6.9%)	14 (23.3%)	15.370 (< 0.001) (0.308)
Sometimes	53 (52.0%)	15 (25.0%)	
Always	42 (41.2%)	31 (51.7%)	
Can feel that I'm going to have an epileptic attack/seizure			
Never	20 (19.6%)	12 (20.0%)	0.514 (0.773) (0.056)
Sometimes	36 (35.3%)	18 (30.0%)	
Always	46 (45.1%)	30 (50.0%)	
Usually try to fight off epileptic seizures			
Never	14 (13.7%)	17 (28.3%)	6.463 (0.040) (0.200)
Sometimes	40 (39.2%)	15 (25.0%)	
Always	48 (47.1%)	28 (46.7%)	
Falling during an epileptic seizure			
Never	9 (8.8%)	6 (10.0%)	20.748 (< 0.001) (0.358)
Sometimes	55 (53.9%)	11 (18.3%)	
Always	38 (37.3%)	43 (71.7%)	
When I have epileptic seizures, I lose consciousness			
Never	12 (11.8%)	8 (13.3%)	3.440 (0.179) (0.146)
Sometimes	49 (48.0%)	20 (33.3%)	
Always	41 (40.2%)	32 (53.3%)	
When I have epileptic seizures, I wet myself			
Never	32 (31.4%)	12 (20.0%)	22.475 (< 0.001) (0.372)
Sometimes	40 (39.2%)	8 (13.3%)	
Always	30 (29.4%)	40 (66.7%)	
Feels weak and sleepy after an epileptic seizure			
Never	9 (8.8%)	2 (3.3%)	18.852 (< 0.001) (0.341)
Sometimes	43 (42.2%)	8 (13.3%)	
Always	50 (49.0%)	50 (83.3%)	
Suffer from headache after an epileptic seizure			
Never	10 (9.8%)	5 (8.3%)	6.293 (0.043) (0.197)
Sometimes	46 (45.1%)	16 (26.7%)	
Always	46 (45.1%)	39 (65.0%)	
Tongue bite during seizures			
Never	26 (25.5%)	5 (8.3%)	14.146 (0.001) (0.295)
Sometimes	45 (44.1%)	20 (33.3%)	
Always	31 (30.4%)	35 (58.3%)	
Experience epileptic attacks in my sleep			
Never	24 (23.5%)	6 (10.0%)	14.563 (0.001) (0.300)
Sometimes	53 (52.0%)	22 (36.7%)	
Always	25 (24.5%)	32 (53.3%)	

Most PWE reported that they try to fight off epileptic seizures. The majority of PWE reported that they fall (*p* > 0.001), lose consciousness (*p* = 0.179), wet themselves (*p* > 0.001), feel weak and sleepy (*p* > 0.001), suffer from headache (*p* = 0.043) and bite tongue (*p* > 0.001). The study also revealed that most PWE experience epileptic seizures in their sleep.

Many PWE reported experiencing antiseizure drug side effects always or sometimes, even though many did not (see Table 4). It is impossible to overlook the number of people suffering from antiseizure drug side effects. Most PWE reported being unable to perform strenuous work or play with their friends due to the antiseizure treatment. Because of the antiseizure treatment, it was also noted that PWE felt tired.

**TABLE 4:** PWE experiences of effects of anti‐seizure medications.

	Province		*χ* ^2^ (*p*‐value) Phi and Cramer's
	Limpopo	Mpumalanga	
Always suffer from the side effects of antiseizure medications			
Never	49 (48.0%)	30 (50.0%)	9.679 (0.008) (0.244)
Sometimes	38 (37.3%)	11 (18.3%)	
Always	15 (14.7%)	19 (31.7%)	
Can't perform strenuous work because of antiseizure treatment			
Never	23 (22.5%)	8 (13.3%)	9.299 (0.010) (0.240)
Sometimes	38 (37.3%)	13 (21.7%)	
Always	41 (40.2%)	39 (65.0%)	
Can't play with my friends because of the antiseizure treatment			
Never	35 (34.3%)	17 (28.3%)	5.337 (0.069) (0.182)
Sometimes	39 (38.2%)	16 (26.7%)	
Always	28 (27.5%)	27 (45.0%)	
Always tired because of antiseizure medication			
Never	39 (38.2%)	21 (35.0%)	8.138 (0.017) (0.224)
Sometimes	39 (38.2%)	13 (21.7%)	
Always	24 (23.5%)	26 (43.3%)	

Those who responded to the questions posed indicated that they faced various issues. Antiseizure treatment resulted in school dropouts and job losses for some due to its adverse effects and the presence of epilepsy. These are evident mainly from the 65.7% (Limpopo Province) and 71.7% (Mpumalanga Province) who either dropped out of primary, secondary or tertiary education as they suffered from adverse effects and other social issues, including stigma and discrimination. According to PWE, epilepsy and antiseizure medication promote dependency on family members or relatives. Some reported suffering from low self‐esteem and experiencing maltreatment, stigma, and discrimination from family members, relatives, and community members.

According to the research, these factors contributed to self‐ or family‐induced isolation, which contributed to PWE's inability to engage with others, which had a psychological impact on the PWE. Others have mentioned feeling depressed, helpless, ashamed, and emotionally stressed.

## 4 DISCUSSION

The main objective of this study was to explore the experiences of individuals living with epilepsy (PWE) undergoing antiseizure treatment in rural South Africa. This study provided valuable insights regarding the frequency of seizures and the impact of epilepsy on the quality of life of those living with the condition. Previous studies reported that epilepsy was usually diagnosed during the first 10 years of life,^25^, ^26^ consistent with the findings from the current study, that the age range for diagnosis was between 0 and 9 years.^27^ Although most participants in the current study reported experiencing occasional seizures, most were proceeded by some form of warning sign. Baumgartner et al.^28^ reported that various warnings, such as a sense of heat or cold, palpitation, sexual arousal, respiratory change, and goosebumps, can proceed seizures. In contrast to the current study, Berg et al.^26^ found that PWE frequently experienced seizures without any warnings. The experience of epileptic seizures varies among PWE, as Fisher et al.^29^ noted, with seizures occurring at different frequencies, ranging from less than one per year to several per day.^29^ The impact of epilepsy is extensive, with unpredictable and mostly hazardous incidences of seizures.^3^, ^8^ Proper diagnosis and treatment can help 70% of PWE to live free of seizures.^3^, ^30^ However, in the current study, over half of PWE reported experiencing occasional seizures, while a significant proportion indicated experiencing frequent seizures. Ayalew and Muche^31^ reported that over half of the patients had between one to five seizures in the previous year, with 22.3% experiencing one or two seizures at their most recent follow‐up. These findings suggest that seizure control is lacking among patients despite the use of antiseizure treatment. Factors such as a lack of availability of ASM in low‐income countries, identified by WHO,^3^ may contribute to the continuation of seizure episodes among patients.

The results from this study show that seizures significantly affect PWE, producing a loss of consciousness, headaches, weakness, tongue bite, and sleeping disturbances, consistent with a systematic review by Subota et al.^32^ Several studies, including Nagabushana et al.,^33^ Tsvere et al.,^17^ and Lee,^34^ have reported that antiseizure treatment can have various side effects, such as psychological, general, and social effects. Dizziness, fatigue, and stomach upset are common adverse reactions reported by PWE in this study, affecting their ability to make friends and engage in strenuous activities. This finding is consistent with Ayalew and Muche's^31^ report that patients experience fatigue due to antiseizure treatment. The study's results also revealed that PWE faces difficulties playing with others, consistent with findings by Fazeka et al.^35^ Asahngwa et al.^36^ reported that epilepsy negatively affects PWE social relationships, leading to psychological, economic, and emotional distress in their daily lives and activities. Fazeka et al.^35^ emphasized that seizures affect PWE mentally and physically, leading to various limitations in their daily life.

The impact of antiseizure treatment on PWE is well‐documented, including its effects on self‐esteem, stigma, dependency, isolation, attention, and discrimination.^17^, ^34^, ^35^ In the current study, PWE reported feeling dependent, depressed, and helpless due to antiseizure treatment. They also highlighted that self‐ or family‐induced isolation contributed to their inability to engage with others due to their medication. Additionally, the study found that a majority of PWE are not married, which is consistent with previous research.^36^, ^37^, ^38^, ^39^

The findings of this study have several practical implications for healthcare providers and policymakers in improving PWE's quality of life. The study highlights the need for the proper diagnosis and treatment of epilepsy, leading to improved seizure control. Healthcare providers must also be aware of the various side effects of antiseizure treatment and address them accordingly. This study also emphasizes the need to address the social and psychological effects of epilepsy on PWE, such as isolation, dependency, and discrimination, and the need for social support systems to be in place. Healthcare providers and policymakers must also consider the impact of epilepsy on PWE ‘s education and employment opportunities and implement measures to ensure that PWE have access to these opportunities. The findings also suggest the importance of promoting awareness and education to the general public about epilepsy to address the negative perceptions and stigma surrounding the condition. Finally, the results of this study highlight the need for further research on epilepsy in LMICs to improve our understanding of the condition and develop effective interventions to improve the quality of life of PWE.

## 5 LIMITATIONS

The main advantage of this study is its capacity to collect information from PWE in two provinces of South Africa. Nonetheless, a drawback is the need for more participants from Mpumalanga, which affected the representation of Swati, Venda, Afrikaans, Zulu, and Sotho‐speaking patients.

## 6 CONCLUSIONS AND RECOMMENDATIONS

Our study's exploration into the experiences of PWE in Limpopo and Mpumalanga reveals critical insights. A significant early diagnosis rate, particularly in children under nine, underscores the urgency for school educational initiatives to foster understanding from a young age. Persistent seizures, despite treatment, signal a pressing need for more research into the efficacy of medication regimes and patient adherence. Our findings illuminate the profound influence of epilepsy and its treatment on life's fundamental aspects, such as education and employment, stressing the imperative for comprehensive support strategies that go beyond medical intervention.

Moving forward, we recommend a multifaceted approach to bolster PWE's quality of life. This should include revamping diagnostic procedures, targeted professional development in rural medical facilities, and widespread educational campaigns to dispel myths and stigma. A concerted effort by government and healthcare systems is essential, aimed at refining care and mitigating epilepsy's toll on daily life. This endeavor must also tackle the intricacies of healthcare access—namely, its availability, affordability, and eliminating barriers to care. A nuanced understanding of individual factors, such as treatment adherence, demographic influences, and personal beliefs, is vital to tailor interventions effectively and ensure they resonate with PWE's needs.

## AUTHOR CONTRIBUTIONS

All Authors contributed to the design, execution, and write‐up of this manuscript. All authors contributed substantially to the conception, design, acquisition, analysis and write‐up, while Lufuno Makhado contributed to the analysis and interpretation of the findings. All authors contributed substantially to the modified versions and published version of the manuscript.

## CONFLICT OF INTEREST STATEMENT

None of the authors has any conflict of interest to disclose. We confirm that we have read the Journal's position on issues involved in ethical publication and affirm that this report is consistent with those guidelines.

## Data Availability

Datasets for the current study are available from the corresponding author by written request to authentic scientific investigators upon release approval.
